# Effects of aminophylline therapy on urine output and kidney function in children with acute kidney injury

**DOI:** 10.1007/s00467-023-06065-y

**Published:** 2023-08-03

**Authors:** Beatrice I. Nyann, Peter Nourse, Adelaide Masu, Kofi Agyabeng, Mignon I. McCulloch

**Affiliations:** 1 Department of Paediatrics, University of Ghana Medical Centre, Legon, Accra, Ghana; 2 Department of Paediatrics and Child Health, University of Cape Town, Cape Town, South Africa; 3 Department of Paediatrics and Child Health, Red Cross War Memorial Children’s Hospital, Rondebosch, Cape Town, South Africa; 4 Department of Biostatistics, School of Public Health, University of Ghana, Legon, Accra, Ghana

**Keywords:** Acute kidney injury, Aminophylline, Urine output, Serum creatinine

## Abstract

**Background:**

Acute kidney injury (AKI) is a frequent complication of children admitted to the paediatric intensive care unit. One key management modality of AKI is the use of diuretics to reduce fluid overload. Aminophylline, a drug that is well known for its use in the treatment of bronchial asthma, is also purported to have diuretic effects on the kidneys. This retrospective cohort study assesses the effect of aminophylline in critically ill children with AKI.

**Methods:**

A retrospective chart review of children admitted to the paediatric intensive care unit of the Red Cross War Memorial Children’s Hospital (RCWMCH) with AKI who received aminophylline (from 2012 to June 2018) was carried out. Data captured and analyzed included demographics, underlying disease conditions, medications, urine output, fluid balance, and kidney function.

**Results:**

Data from thirty-four children were analyzed. Urine output increased from a median of 0.4 mls/kg/hr [IQR: 0.1, 1.1] at six hours prior to aminophylline administration to 0.6 mls/kg/hr [IQR: 0.2, 1.9] at six hours and 1.6 mls/kg/hr [IQR:0.2, 4.2] at twenty-four hours post aminophylline therapy. The median urine output significantly varied across the age groups over the 24-h time period post-aminophylline, with the most response in the neonates. There was no significant change in serum creatinine levels six hours post-aminophylline administration [109(IQR: 77, 227)—125.5(IQR: 82, 200) micromole/l] P-value = 0.135. However, there were significant age-related changes in creatinine levels at six hours post-aminophylline therapy.

**Conclusions:**

Aminophylline increases urine output in critically ill children with AKI.

**Graphical abstract:**

**Figure:**
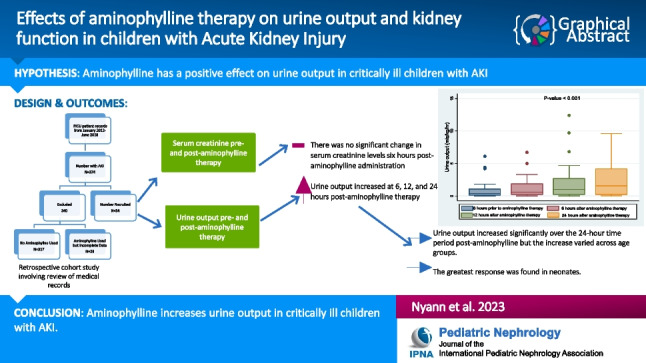
A higher resolution version of the Graphical abstract is available as Supplementary information

**Supplementary Information:**

The online version contains supplementary material available at 10.1007/s00467-023-06065-y.

## Introduction

The incidence of acute kidney injury (AKI) is rising in both high income countries (HIC) and lower or lower middle income countries(LLMIC) and is associated with severe morbidity and mortality especially in children [1]. Hospital studies in LLMIC report AKI in 3.2- 9.6% of admissions with overall in-hospital mortality around 20% and up to 50% in intensive care unit (ICU) patients [2, 3]. Incidence of AKI in critically ill children varies widely in paediatric intensive care units (PICUs) around the world in both LLMIC and HIC and ranges from 25.1% to 82% [4–7].

There have been attempts at pharmacologic treatment of AKI. Agents that have been used include dopamine, vasopressin and fenoldopam but outcomes have not been beneficial [8–10]. These findings may be partly due to the delay in commencement of these therapies premised on the imprecision of current AKI biomarkers. A crucial part of the conservative management of AKI involves fluid balance. Maintaining a neutral fluid balance remains a significant challenge especially in critically ill children with AKI where fluid replacement and fluid overload must be delicately balanced. Several paediatric and adult studies have demonstrated a positive relationship between fluid overload and mortality in critically ill patients with AKI [11, 12]. Alobaidi et al. [13] in their large systematic review of 44 studies and 7507 children highlighted this phenomenon. In addition, the use of kidney replacement therapy (KRT) can be limited by managing fluid overload with diuretics.

Aminophylline is one drug identified to be very promising in this regard. Aminophylline is a xanthine derivative and the ethylenediamine salt of theophylline. It has long been well known for its use in the treatment of airway disease by the facilitation of bronchodilatation [14, 15]. However, in addition to aminophylline’s use in the treatment of respiratory conditions including asthma [16], it has been demonstrated to have diuretic and reno-protective effects [17–19]. Aminophylline achieves this effect by non-specifically blocking adenosine receptors and inhibiting their vasoconstrictive effects [20]. Aminophylline therefore has been purported to improve urine output in AKI [21]. A meta-analysis of the use of aminophylline in contrast-induced nephropathy demonstrated an improvement in the eGFR and increased creatinine clearance with the use of aminophylline [22]. Tamburro et al. [23], in their single-centre retrospective study demonstrated that urine output increased significantly with aminophylline use in the thirty-five patients studied [median increase 0.5 ml/kg/h (IQR: -0.3, 1.3), P-value = 0.05]. There is therefore increasing evidence for aminophylline use as an adjunct diuretic in AKI.

Most studies have focused on the use of aminophylline in specific conditions notorious for being complicated by AKI, namely: birth asphyxia, cardiopulmonary bypass for cardiac surgery and contrast-induced nephropathy [24–28]. This study brings to the fore the effect of aminophylline on AKI as a result of varied conditions not limited to the above-mentioned.

We assessed the effect of aminophylline administration in children with AKI in the PICU of Red Cross War Memorial Children’s Hospital (RCWMCH). The objectives were to determine the effect of aminophylline on urine output and kidney function (serum creatinine).

## Primary and secondary outcomes

The primary outcome was mean urine output (ml/kg/hr) at 6 and 24 h post-aminophylline administration. Secondary outcomes were the difference in means of serum creatinine at 6 h pre- and post-aminophylline therapy, and the relationship between age and response to aminophylline therapy.

## Methods

### Study design and population

**Fig. 1:**
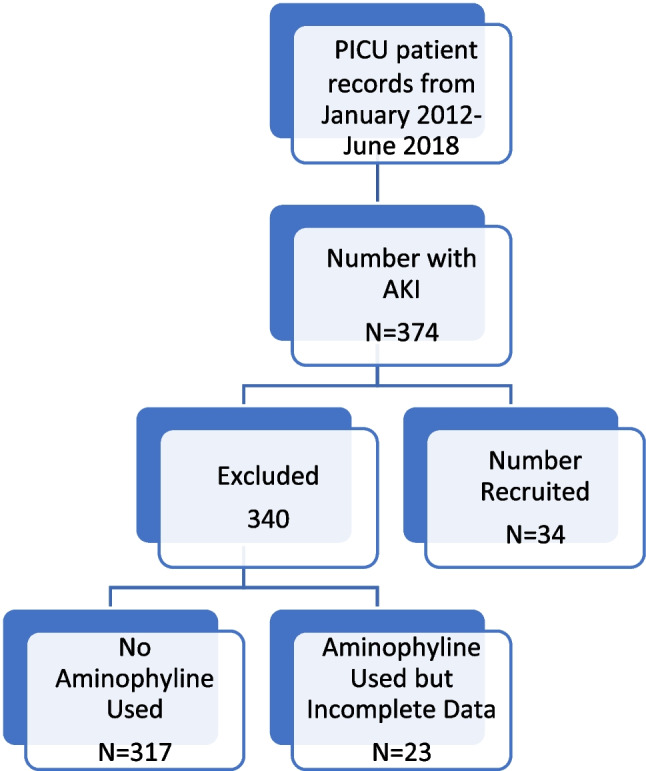
Participant selection process

We carried out a retrospective cohort study involving a review of the medical records of children from birth to fifteen years with AKI as per the Kidney Disease Improving Global Outcomes (KDIGO) criteria who were admitted to the PICU of RCWMCH from January 2012 to 1 June 2018 and received aminophylline. Aminophylline use in the PICU of RCWMCH is typically the last conservative resort to augment urine output in children with AKI. Exclusion criteria included children with known pre-existing kidney disease, diabetes insipidus, those who had aminophylline less than two hours after starting furosemide as well as those with significant incomplete data. All participants recruited received at least 24 h of aminophylline at a dose of 1 mg/kg body weight given as an intravenous bolus every 6 h and had no new diuretic added during the 24-h period. All recruited participants were on furosemide at least two hours before the start of aminophylline. The RCWMCH is located in the Western Cape with a bed capacity of 300 and has a very well equipped 39-bed capacity ICU which admits on average 115 patients per month (Fig. 1).

### Definition of variables

#### AKI staging

The AKI stage of children recruited was categorized according to the 2012 KDIGO AKI guidelines [29].

#### PIM3 score

This is one of the severity scoring systems used for predicting the outcome of paediatric patients admitted to the PICU based on data collected within the first hour of admission [30].

#### Data collection

All children admitted to the PICU with AKI as per the KDIGO AKI guidelines and who were on aminophylline were retrospectively recruited through a search of the RCWMCH PICU database and a chart review. A manual review of discharge summaries of all patients admitted to the PICU in the specified time period (January 2012 to June 2018) was carried out with subsequent recruitment of patients with AKI as part of their diagnoses who had aminophylline for at least 24 h. Using a data extraction tool, demographic data, PIM3 scores, diagnoses, medications, ventilatory status and weight were obtained from the recruited patients’ records. Urine output (obtained via indwelling urethral catheters) for these times were abstracted from the patients’ records and recorded as milliliters per kilogram per hour (mls/kg/hr), 6 h prior to aminophylline therapy, and 6, 12 and 24 h after aminophylline therapy. Six hours pre- and post-aminophylline administration electrolytes, urea and creatinine were also obtained from the patients’ records. For parameters not done exactly six hours pre- and post-aminophylline therapy the closest to the required six-hour periods were used. Other outcomes of interest that were obtained from medical records included fluid balance (percentage fluid overload calculated), the total duration of AKI in days, the length of PICU stay and the overall outcome in PICU (recovery of kidney function, defined by normalization of serum creatinine by hospital discharge or three months post-discharge or not, or mortality). Percentage fluid overload was calculated using the formula $\frac{total\;fluid\;input-fluid\;output}{admission\;weight\;in\;kg}\times {100}$ [31].

### Statistical analysis

Data was entered into a spreadsheet (Microsoft Excel) and imported into STATA version 15 for data management and analysis. All numerical continuous variables of interest were represented by means with standard deviation, median and interquartile ranges (IQR) based on the distribution of that data. All categorical data were represented in terms of frequencies and percentages. Friedman’s Analysis of Variance test was used in assessing urine output across four time points (6 h prior, 6,12, and 24 h post start of aminophylline therapy). Wilcoxon signed-rank test and paired t-test were used in comparing serum creatinine at 6 h pre- and 6 h post-aminophylline therapy. Quantile regression with robust and clustered standard errors model was used to assess the effect of time on the various outcomes of interest. All statistical tests were done at 5% level of significance.

#### Ethical approval

Ethical approval was obtained from the University of Cape Town (UCT) Faculty of Health Sciences Research Ethics Committee. Ethics approval number: 603/2018.

#### Consent to participate

A waiver of consent was approved by the University of Cape Town Faculty of Health Science Research Ethics Committee.

## Results

### Background characteristics of study participants

**Fig. 2:**
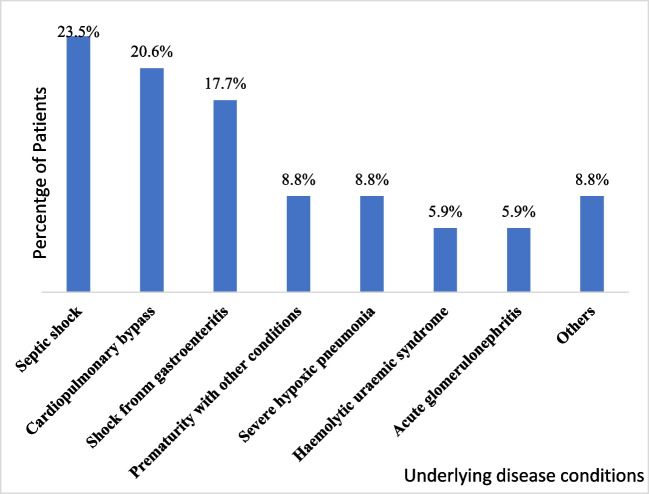
Underlying causes of AKI in patients

Data on thirty-four children were available for analysis. The median age of the children was three months with a quarter of them being less than one month old and three-quarters of them aged 15 months. Females were the predominant sex (52.9%, 18/34). About two-thirds of the children had Stage 3 AKI according to the KDIGO classification. Thirty of the children (88.2%,30/34) were on ventilatory support. Among those on ventilatory support, mechanical ventilation was the most common mode used (80.0%, 24/30). On average, PIM3 level among the children was -2.33 ± 1.60. This translates to average mortality risk of 8.47% (1.8% to 31.86%). The children had a median weight of 4.90 kg (IQR 2.50, 10.00) (Table 1). The most common diagnoses included septic shock (20.5%, n = 7), cardiopulmonary bypass (20.5%, n = 7) and shock from gastroenteritis (17.6%, n = 6) (Fig. 2). About three of every ten selected children died (28.6%, 10/34). This mortality rate was higher than the average predicted by the PIM3 score, though it falls within the range predicted by the PIM3 score.

**Table 1:** Background characteristics of study participants

	Frequency	Percentage
Age in months:		
Median (UQ, UL)	3.25 (0.75, 15.00)	
< 1 month	11	32.35
1–5.9 months	10	29.41
6-48 months	7	20.59
> 48 months	6	17.65
Sex		
Male	16	47.06
Female	18	52.94
Staging		
Stage 1	1	2.94
Stage 2	8	23.53
Stage 3	25	73.53
Ventilatory Support		
No	5	14.71
Yes	29	85.29
Type and ventilatory support		
Mechanical ventilation	23	79.31
CPAP	4	13.79
Other	2	6.90
PIM3: Mean ± SD	–2.33 ± 1.60	
Weight: Median (UQ, UL)	4.90 (2.50, 10.00)	
Medications (multiple response)		
Furosemide	34	100.00
Inotropes	28	82.35
Antibiotics	33	94.12
Recovery of kidney function		
No	21	61.76
Yes	13	38.24
Mortality		
No	24	70.59
Yes	10	29.41

*LQ* Lower Quartile; *UQ* Upper Quartile; *CPAP* Continuous positive airway pressure; *PIM3* Paediatric index of mortality

### Urine output

**Fig. 3:**
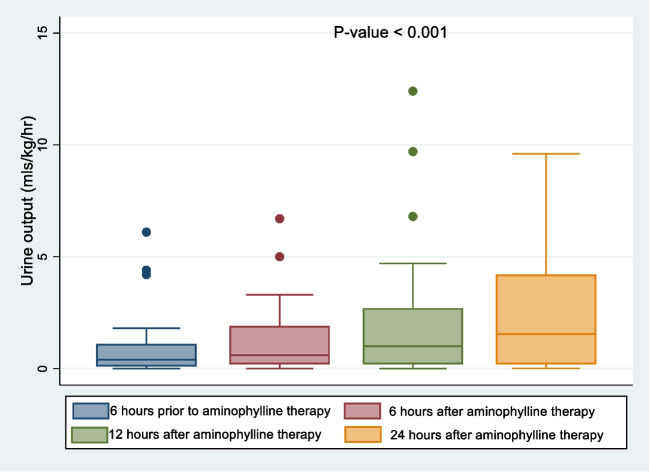
Urine output of children 6 h prior and 6, 12, 24 h post-aminophylline therapy

Thirty-four patients had pre-and post-aminophylline urine output assessed; urine output increased from a median of 0.4 mls/kg/hr (IQR:0.1, 1.1) at six hours prior to aminophylline therapy to 0.6 mls/kg/hr (IQR:0.2, 1.9) after six hours, 1.0 mls/kg/hr (IQR:0.2, 2.7) after twelve hours and 1.6 mls/kg/hr (IQR:0.2, 4.2) after twenty-four hours post-aminophylline therapy (p-value = 0.001) (Fig. 3). The median change in post-aminophylline urine output after six hours was 0.05 mls/kg/hr ([IQR:0.0, 0.6], p-value = 0.015). The median urine output at 6 h, 12 h and 24 h post-aminophylline was significantly greater than urine output at six hours prior to aminophylline treatment.

Comparatively, the median urine output significantly varied across the age groups and over time. Babies aged < 1 month had the highest increase in urine output while those aged 6–48 months and > 48 months had the least increase in urine output. The median urine output decreased with higher age group (Table 2).

**Table 2:** Urine output over time by participant characteristics

	6 h Prior	6 h after	12 h after	24 h after	
	Median (LQ, UQ)	Median (LQ, UQ)	Median (LQ, UQ)	Median (LQ, UQ)	*P*-value*
Total	0.36 (0.10, 0.90)	0.55 (0.20, 1.70)	0.85 (0.20, 2.70)	1.40 (0.20, 4.00)	< 0.001
Age					< 0.001
< 1 month	0.40 (0.30, 4.20)	1.00 (0.04, 1.90)	1.80 (0.24, 2.70)	2.70 (0.20, 7.00)	
1–5.9 months	0.30 (0.05, 0.80)	0.45 (0.30, 1.90)	0.60 (0.30, 2.80)	2.60 (0.30, 3.80)	
6-48 months	0.80 (0.22, 0.90)	1.25 (0.10, 2.50)	1.40 (0.05, 4.00)	1.40 (0.05, 5.60)	
> 48 months	0.25 (0.02, 0.40)	0.35 (0.20, 0.90)	0.20 (0.20, 1.00)	0.45 (0.10, 0.50)	
Sex					0.131
Male	0.45 (0.16, 1.30)	0.55 (0.15, 1.70)	1.10 (0.13, 2.70)	1.65 (0.13, 3.35)	
Female	0.31 (0.09, 0.90)	0.70 (0.30, 1.70)	0.80 (0.24, 2.00)	1.40 (0.30, 4.20)	
AKI Stage					< 0.001
Stage 1	0.80 (0.80, 0.80)	2.50 (2.50, 2.50)	4.00 (4.00, 4.00)	2.10 (2.10, 2.10)	
Stage 2	0.70 (0.31, 1.33)	1.18 (0.75, 2.00)	2.10 (0.85, 2.85)	2.80 (1.25, 6.30)	
Stage 3	0.30 (0.09, 0.80)	0.40 (0.10, 1.50)	0.60 (0.10, 1.80)	0.80 (0.10, 3.90)	
Ventilator support					0.036
No	0.30 (0.20, 0.40)	0.60 (0.01, 0.90)	1.00 (0.10, 1.50)	1.10 (0.40, 2.70)	
Yes	0.40 (0.10, 1.10)	0.50 (0.20, 1.70)	0.70 (0.20, 2.70)	1.55 (0.16, 4.10)	
Inotropes					0.136
No	0.60 (0.20, 1.30)	1.30 (0.40, 3.30)	1.30 (0.60, 3.00)	0.90 (0.10, 3.20)	
Yes	0.36 (0.10, 0.85)	0.50 (0.15, 1.50)	0.65 (0.20, 2.50)	1.70 (0.20, 4.20)	
Antibiotics					< 0.001
No	0.35 (0.20, 0.50)	0.95 (0.90, 1.00)	1.85 (1.00, 2.70)	1.40 (0.40, 2.40)	
Yes	0.36 (0.10, 1.00)	0.50 (0.15, 1.80)	0.65 (0.20, 2.50)	1.40 (0.12, 4.20)	

*LQ* Lower Quartile; *UQ* Upper Quartile

### Fluid balance and kidney function

The median fluid balance did not vary significantly over the study time period. The percentage of fluid overload varied significantly over the study time period. At 6 h prior to aminophylline the median fluid overload was 2.46% and decreased consistently over time to -0.17% at 24 h post-aminophylline treatment. Serum urea, creatine, potassium, sodium and chloride levels were assessed six hours before and six hours after aminophylline treatment. There was no statistically significant change in serum creatinine levels six hours after treatment (Table 3).

**Table 3:** Fluid overload and kidney function of participants pre- and post-aminophylline therapy

Variable	6 h Prior	6 h after	12 h after	24 h after	*P*-value
	Median (LQ, UQ)	Median (LQ, UQ)	Median (LQ, UQ)	Median (LQ, UQ)	
Fluid overload					
Fluid Balance	76.00 [30.00, 223.00]	96.00 [8.00, 159.50]	101.30 [–8.00, 228.00]	21.60 [–85.00, 97.55]	0.270
% Fluid overload	2.46 [0.50, 5.14]	2.00 [0.40, 4.42]	1.60 [–0.32, 5.34]	–0.17 [–1.83, 1.66]	0.007**
Kidney function					
Serum urea^1^	13.1 (7.5, 23.0)	14.85 (8.9, 22.0)	–	–	0.017*
Serum creatinine^2^:	109 (77, 227)	125.5 (82, 200)	–	–	0.135
Serum potassium^1^: Mean ± SD	4.65 ± 1.16	4.14 ± 0.75	–	–	0.018*
Serum sodium^1^: Mean ± SD	138.26 ± 5.9	138.71 ± 5.64	–	–	0.534
Serum chloride^1^:	106 (98, 110)	104.5 (100, 107)	–	–	0.178

*LQ* Lower Quartile; *UQ* Upper Quartile; 1, mmol/L; 2, umol/L

*p < 0.05, **p < 0.01, ***p < 0.001

The median serum creatine levels significantly varied across the age groups over time. Babies aged 1–5.9 months and 6–48 months had a higher increase in serum creatinine level than those aged < 1 month, and those aged > 48 months had the least variation in serum creatinine (Table 4).

**Table 4:** Serum creatinine over time by participant characteristics

	Serum Creatinine		
	6 h Prior (LQ, UQ)	6 h after (LQ, UQ)	*P*-value*
Total	112.00 (77.00, 227.00)	131.00 (87.00, 200.00)	0.197
Age			< 0.001
< 1 month	96.00 (58.00, 161.00)	105.00 (60.00, 165.00)	
1–5.9 months	96.00 (32.00, 106.00)	109.00 (82.00, 137.00)	
6-48 months	118.00 (94.00, 310.00)	140.00 (114.00, 386.00)	
> 48 months	526.50 (137.00, 754.00)	536.00 (150.00, 718.00)	
Sex			< 0.001
Male	105.50 (82.00, 143.00)	107.00 (74.00, 166.50)	
Female	129.00 (77.00, 535.00)	156.00 (108.00, 541.00)	
AKI Stage			0.813
Stage 1	118.00 (118.00, 118.00)	114.00 (114.00, 114.00)	
Stage 2	102.00 (60.00, 161.00)	120.00 (60.00, 165.00)	
Stage 3	112.00 (94.00, 310.00)	137.00 (96.00, 366.00)	
Ventilatory support			0.983
No	535.00 (129.00, 702.00)	557.00 (156.00, 690.00)	
Yes	105.50 (65.00, 182.00)	117.00 (78.00, 191.00)	
Inotropes			0.869
No	422.50 (102.00, 702.00)	471.50 (42.00, 690.00)	
Yes	106.00 (70.00, 161.00)	120.00 (87.00, 183.00)	
Antibiotics			0.963
No	378.00 (54.00, 702.00)	375.50 (61.00, 690.00)	
Yes	112.00 (77.00, 227.00)	131.00 (87.00, 200.00)	

*LQ* Lower Quartile; *UQ* Upper Quartile

## Discussion

The children involved in the study were around three months of age with more than half of them being females. The study found a significant increase in urine output six and twenty-four hours post-aminophylline therapy. This change in urine output varied significantly across the age groups with time after controlling for participant characteristics including inotrope use. There was no statistically significant change in serum creatine post-aminophylline therapy, albeit significant age-related variation in serum creatinine with time.

Various studies over the years have evaluated therapeutic interventions for the prevention and treatment of AKI including the use of aminophylline [29–33]. However, only a few of these studies were conducted in sub-Saharan Africa [19]. This retrospective cohort study is one of the few in sub-Saharan Africa to evaluate the effects of aminophylline in acute kidney injury. This study has demonstrated that aminophylline increases urine output in AKI. Most studies have concentrated on specific cases that are at risk of AKI, for example neonates with birth asphyxia [24, 25], whereas our study has analyzed different age groups of children with various underlying conditions.

The use of aminophylline to improve urine output in AKI, though not its prime therapeutic use, is not entirely novel and has been reviewed by some studies [19, 23, 32]. Methylxanthines both natural and synthetic are known to cause diuresis by inhibiting sodium reabsorption in the proximal tubules through blockade of adenosine A1 receptors [33]. They also cause renal vasodilatation by competitively antagonizing adenosine-induced constriction of the afferent arteriole and reducing efferent arteriolar vasoconstriction to some degree and hence improve renal perfusion [33]. Prior to the advent of more potent diuretics, both aminophylline and theophylline were used in critically ill children with fluid overload [34]. This study confirmed the positive effect aminophylline has on urine output. There was a statistically significant increase in urine output from a median of 0.4 mls/kg/hr [IQR:0.1,1.1] at 6 h prior to administration of aminophylline to 0.6 mls/kg/hr [IQR: 0.2,1.9] at six hours and 1.6 mls/kg/hr [IQR:0.2, 4.2] at 24 h after aminophylline administration. This finding is similar to that found by a number of studies which predominantly looked at the prevention of AKI after cardiac surgeries [28, 35]. In our study, the concomitant use of furosemide could have confounded the positive effect aminophylline had on urine output. However, being a case-crossover study, urine output was assessed before and after the introduction of aminophylline. Aminophylline levels in serum peak at 30 min after intravenous administration and have a half-life of 3 to 30 h. Whereas furosemide after intravenous administration has an onset of action of 5 min, peaks at 30 min and lasts 2 h. In all the study participants furosemide was commenced at least two hours before aminophylline, hence a good baseline for assessing urine output after aminophylline administration. In addition, there is no documented evidence of a synergistic effect between furosemide and aminophylline and as such the effect of each one is independent of the other. Though it has been postulated that singular agents including methylxanthines may have salient effects in the presence of other agents such as antioxidants (eg N-acetylcysteine) [36], none of the participants reviewed were on such agents. However, some participants were on medications that could have altered the serum concentration of aminophylline by either increasing or decreasing its metabolism, for example macrolide antibiotics, ciprofloxacin, phenytoin and rifampicin. The natural progression of AKI may also have played a role in the statistically significant increase in urine output. Another limitation to attributing the increase in urine output solely to aminophylline is the concomitant administration of vasoactive medications which could have contributed to this augmentation in urine output via improvement in blood pressure and subsequently renal perfusion.

Additionally, the increase in urine output varied significantly across the age groups over time. Participants less than 1 month of age had the highest increase in urine output over the twenty-four hour period (6, 12 and 24 h) after administration of aminophylline. The reason for this finding, though not certain, corroborates the postulation that renal tissue response to aminophylline may be age-dependent, having a higher response in neonates [37]. Further controlled studies are imperative in order to make definitive suggestions from this finding.

There is increasing evidence of the negative impact of fluid overload in the critically ill patient. In the recent 22^nd^ Acute Disease Quality Initiative (ADQI) guidelines, it was strongly suggested that all paediatric patients at risk for AKI should have careful assessment of their fluid status involving among other parameters daily fluid balance [37]. Our study found no significant variation in participants’ fluid balance over the study period but there was a statistically significant decrease in the percentage fluid overload of participants from a median of 2.64% at six hours prior to aminophylline therapy to a median of negative 0.17% at 24 h post-aminophylline therapy. This is most likely the product of the significant increase in urine output over the time period, reasons for which have been aforementioned. Aminophylline thus has prospects in the management of fluid overload.

The lack of effect of aminophylline/theophylline on serum creatinine levels has been shown by a number of studies [23, 38] and this is in tandem with what our study found. Improvements in urine output as a result of aminophylline-mediated afferent arteriolar vasodilatation and efferent vasoconstriction leading to an increase in GFR should have been accompanied by an increase in creatinine excretion and hence a drop in the serum creatinine but this was not evidenced by our study. Changes in serum creatine are typically delayed. A longer study duration is warranted to evaluate aminophylline’s effect on serum creatinine levels. Two randomized control trials, all in neonates with birth asphyxia, on the other hand have found the methylxanthines (theophylline, aminophylline) to improve glomerular function and increase creatinine clearance [25, 26]. Thus, the score card on the effect of aminophylline on serum creatinine is mixed.

This index study has brought to the fore the effect of aminophylline regarding AKI, notably improvement in urine output and the marked effect in the neonatal age group. However, the study has significant limitations that necessitate the interpretation of these findings in the context of the study limitations. It was a retrospective descriptive study with no room for controlled interventions and a number of potential study data points were excluded due to incomplete data and the fact that patient selection was based on the diagnosis written in discharge summaries which may not include the diagnosis of AKI. There was also concurrent administration of other diuretics, notably furosemide and in a few participants spironolactone, prior to the start of aminophylline which could have contributed to some of the purported effects of aminophylline or potentiated the effects of aminophylline. Also, other concurrent interventions and treatments, namely fluid therapy, sodium bicarbonate and vasoactive medication administration could have contributed to some of the outcomes. Additionally, some participants were on medication that could potentially interfere with the metabolism of aminophylline and hence its action. In light of the aforementioned, conclusions from this study have been made cautiously due to its limitations.

## Conclusion

Our study has shown an improvement in urine output with aminophylline therapy. However, no significant effect was found on serum creatinine levels with the use of aminophylline. The effect of augmentation of urine output was more pronounced in the neonatal group. Further studies are required to justify these findings due to the small sample size and uncontrolled nature of this study. Despite these limitations aminophylline should be considered as an adjunct diuretic in children with AKI.

### Supplementary Information

Below is the link to the electronic supplementary material.

Graphical abstract (PPTX 58 KB)

## Data Availability

The datasets generated during this study are available from the corresponding author on reasonable request.
