# Supplementary material for: Effects of aminophylline therapy on urine output and kidney function in children with acute kidney injury
Source: Pediatr Nephrol. 2023 Aug 3;39(2):559–67. doi: 10.1007/s00467-023-06065-y (PMC10728232; doi:10.1007/s00467-023-06065-y)
Supplement: Supplementary file 1 — Graphical abstract (PPTX 58 KB) [file 467_2023_6065_MOESM1_ESM.pptx]

## Slide 1
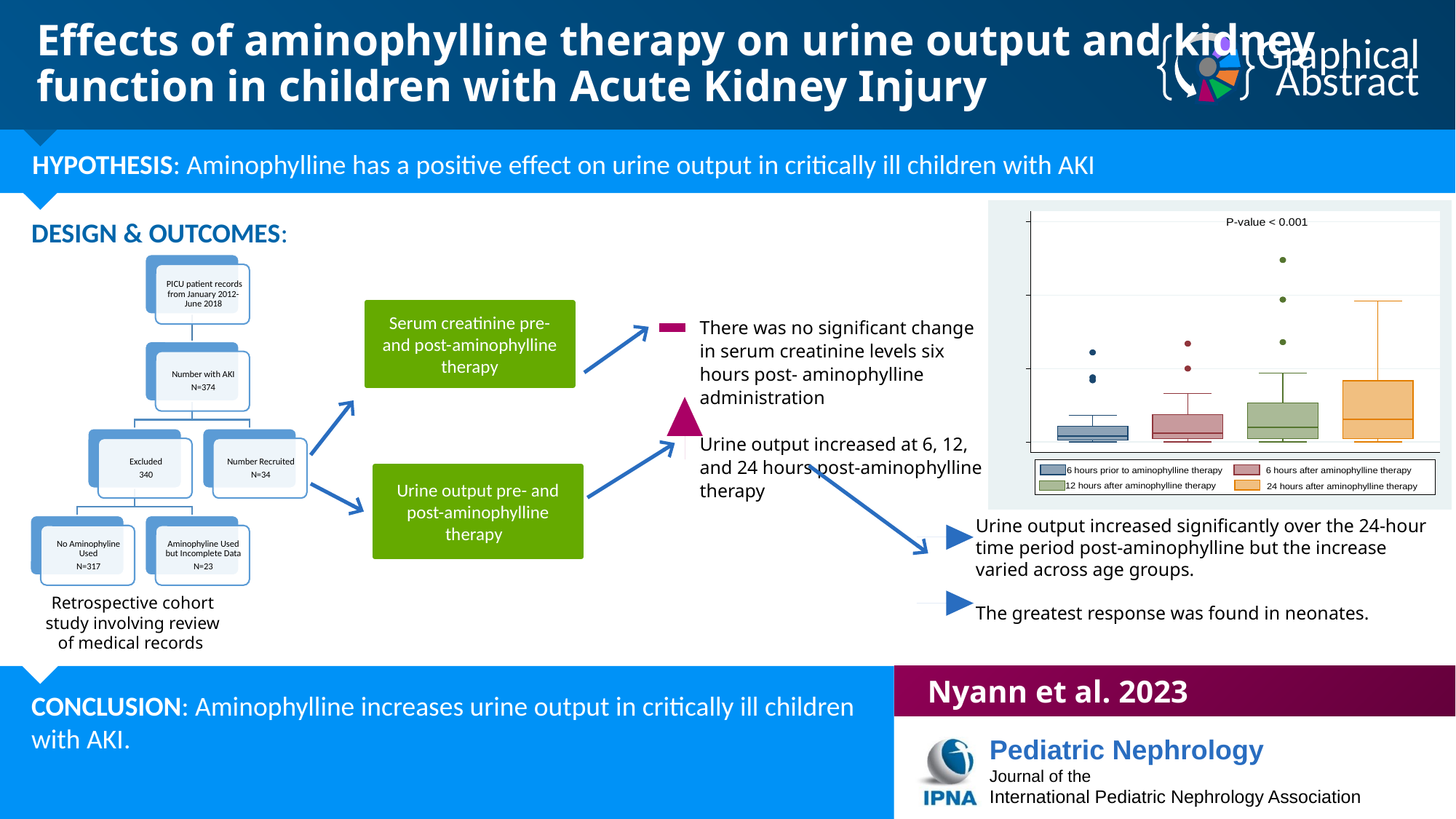

Effects of aminophylline therapy on urine output and kidney
function in children with Acute Kidney Injury
HYPOTHESIS: Aminophylline has a positive effect on urine output in critically ill children with AKI
DESIGN & OUTCOMES:
Serum creatinine pre- and post-aminophylline therapy
There was no significant change in serum creatinine levels six hours post- aminophylline administration
Urine output increased at 6, 12, and 24 hours post-aminophylline therapy
Urine output pre- and post-aminophylline therapy
Urine output increased significantly over the 24-hour time period post-aminophylline but the increase varied across age groups.
The greatest response was found in neonates.
Retrospective cohort study involving review of medical records
Nyann et al. 2023
CONCLUSION: Aminophylline increases urine output in critically ill children with AKI.
